# Speech Discrimination in Real-World Group Communication Using Audio-Motion Multimodal Sensing

**DOI:** 10.3390/s20102948

**Published:** 2020-05-22

**Authors:** Takayuki Nozawa, Mizuki Uchiyama, Keigo Honda, Tamio Nakano, Yoshihiro Miyake

**Affiliations:** 1 Research Institute for the Earth Inclusive Sensing, Tokyo Institute of Technology, Tokyo 152-8550, Japan; 2 School of Computing, Tokyo Institute of Technology, Yokohama 226-8502, Japan; uchiyama.m.ac@m.titech.ac.jp (M.U.); honda.k.al@m.titech.ac.jp (K.H.); 3 Institute for Liberal Arts, Tokyo Institute of Technology, Tokyo 152-8550, Japan; tamio.nakano@me.com; 4 Department of Computer Science, Tokyo Institute of Technology, Yokohama 226-8502, Japan; miyake@c.titech.ac.jp

**Keywords:** speech discrimination, group communication, physical motion, multimodal sensing, sensor fusion, smartphone

## Abstract

Speech discrimination that determines whether a participant is speaking at a given moment is essential in investigating human verbal communication. Specifically, in dynamic real-world situations where multiple people participate in, and form, groups in the same space, simultaneous speakers render speech discrimination that is solely based on audio sensing difficult. In this study, we focused on physical activity during speech, and hypothesized that combining audio and physical motion data acquired by wearable sensors can improve speech discrimination. Thus, utterance and physical activity data of students in a university participatory class were recorded, using smartphones worn around their neck. First, we tested the temporal relationship between manually identified utterances and physical motions and confirmed that physical activities in wide-frequency ranges co-occurred with utterances. Second, we trained and tested classifiers for each participant and found a higher performance with the audio-motion classifier (average accuracy 92.2%) than both the audio-only (80.4%) and motion-only (87.8%) classifiers. Finally, we tested inter-individual classification and obtained a higher performance with the audio-motion combined classifier (83.2%) than the audio-only (67.7%) and motion-only (71.9%) classifiers. These results show that audio-motion multimodal sensing using widely available smartphones can provide effective utterance discrimination in dynamic group communications.

## 1. Introduction

In the various organizations we belong to, we go about our daily lives and build relationships with others through communication. For example, in companies, smooth communications reduce meeting durations and improve work efficiency and productivity, and several studies have proposed effective indicators that lead to such organizational improvement [1,2,3]. Organizational improvement is effective in schools as well as in companies, and it can be utilized for the evaluation and improvement of participatory classes that are recently increasing.

To facilitate communications, it is necessary to first evaluate communications, and utterance discrimination that determines whether a participant is speaking for a particular time is essential in evaluating group communications. However, it is laborious and time-consuming to manually segment speech from recorded audio or video. Therefore, automatic and semi-automatic speech segmentation methods have been actively studied [4,5]. Voice activity detection (VAD), or speech activity detection (SAD), is the fundamental step for these endeavors, which aim to determine “when speech occurred”, i.e., to distinguish time segments that contain speech from those that do not [6]. Although most VAD methods are formulated on a single target speech source, recent studies expanded to multi-speaker VAD in situations with multiple simultaneous speakers [7,8]. The aims of this field also partly overlap with those of research on speaker diarization, which is defined as the task of determining “who spoke when”. Speaker diarization has been applied to multi-speaker speech recognition, generation of meeting minutes, and subtitle generation, and has become prominent in the domain of speech research [9,10]. Speaker diarization systems for specific situations can assume some prior knowledge, such as the number, position, or even identity of speakers present. However, a more ideal general-purpose speaker diarization system is desired to perform speech and non-speech detection, speech segmentation, and speaker identification without specific prior knowledge. Wide use of speaker diarization started with analysis of telephone conversations and broadcast news [10], and has recently extended to the conference meeting domain [9]. Most studies of multi-speaker SAD/VAD and speaker diarization use only audio information for discrimination [8,11,12].

In broadcast news, a single speaker is assumed, and boom or lapel microphones are often used. On the other hand, conference meetings containing multiple speakers are often recorded by desktop or far-field microphones; thus, they are more prone to background noise. Furthermore, unlike broadcast news, there are no prepared manuscripts in conference settings, resulting in frequent overlapped speech. Such situations cause errors in utterance discrimination, especially in cases where a speaker talks more quietly than others, or multiple people talk simultaneously [13]. It is also argued that the performance of audio-only algorithms may have plateaued [14], and studies using visual sensing instead of audio sensing [13], or studies on multimodal approach, are attracting attention. In particular, studies using visual information in addition to audio are increasing. To improve the performance under the circumstances described above, studies have focused on non-verbal cues such as the lip, facial, or hand motions of speakers [7,14,15,16]. Moreover, non-verbal cues often accompany speech, and more studies have investigated the inherently meaningless gestures that occur naturally during speech [17]. Other studies have revealed that events such as turn-taking, listening, or interruption in speech are accompanied by different types of gestures or body movement [18,19,20].

In comparison to the use of visual sensing, the use of physical motion sensing with a wearable sensor is another possible direction for facilitating speech discrimination by capturing physical motions accompanied by speech. Studies have been conducted on speech recognition using the physical activity information obtained from accelerometers [21] and on identifying characteristic motion rhythm and gesture frequencies while talking/listening [1]. Thus, in utterance discrimination, the multimodal approach of combining audio and motion information is expected to increase discrimination accuracy. In addition, in situations where a large number of people participate and change their positions dynamically, it is difficult to cover them by visual sensing using cameras; hence, the use of wearable sensors is expected to be particularly useful in these situations. Moreover, smartphones are widely owned and used, and they are equipped with acceleration and audio sensors. This has led to a growth in the field of human activity recognition using wearable sensors [22,23,24,25]; hence, by using smartphones as wearable sensors, speech discrimination can become easily accessible anywhere by merely installing an application. However, this hypothesis has not been explicitly tested.

Therefore, in this study, we aimed to verify the hypothesis that in group communications consisting of multiple speakers, higher-accuracy discrimination can be achieved by combining the audio and physical motion data obtained by smartphones used as wearable sensors, in comparison to unimodal utterance discrimination using only audio data. The target task can be positioned as multi-speaker SAD/VAD for people wearing smartphone sensors. With the data recorded simultaneously by using multiple smartphone sensors in a participatory class at a university where a large number of people formed groups and communicated simultaneously, we tested the following three-step hypotheses: (1) the physical motion data we focused on in this study contains information about occurrence and absence of speech; (2) in the utterance discrimination within individuals, where individual differences are insignificant, higher accuracy can be achieved by using both the audio and physical motion information, in comparison to using only the audio information; and (3) in comparison to using only audio data, the combined usage of the multimodal data can also benefit the utterance discrimination over individuals, where an existing model trained with a fixed set of data is desired to perform well for new data without requiring the labor of manual speech annotation for the new data.

## 2. Materials and Methods

### 2.1. Participants

University students taking a participatory class that aimed to cultivate communication and facilitation skills participated in the experiment. Throughout the course, the students experienced various kinds of communication practices, such as self-introduction, interview, dialogue, presentation, and facilitation of a small group through a workshop. Data from 12 Japanese students (three females and nine males) from three groups measured in the final class of the term were used for this study. In this final class, the students had small group discussions about the important points that they had learned throughout the course for successful communication, the goals and aspirations they currently held, how they could make use of what they had learned in striving for those goals, and their suggestions for improving the participatory class. All the communication was in Japanese.

Written informed consent was obtained from all participants. The study was approved by the Ethics Committee of Tokyo Institute of Technology and was conducted in accordance with the Declaration of Helsinki.

### 2.2. Data Acquisition

**Figure 1:**
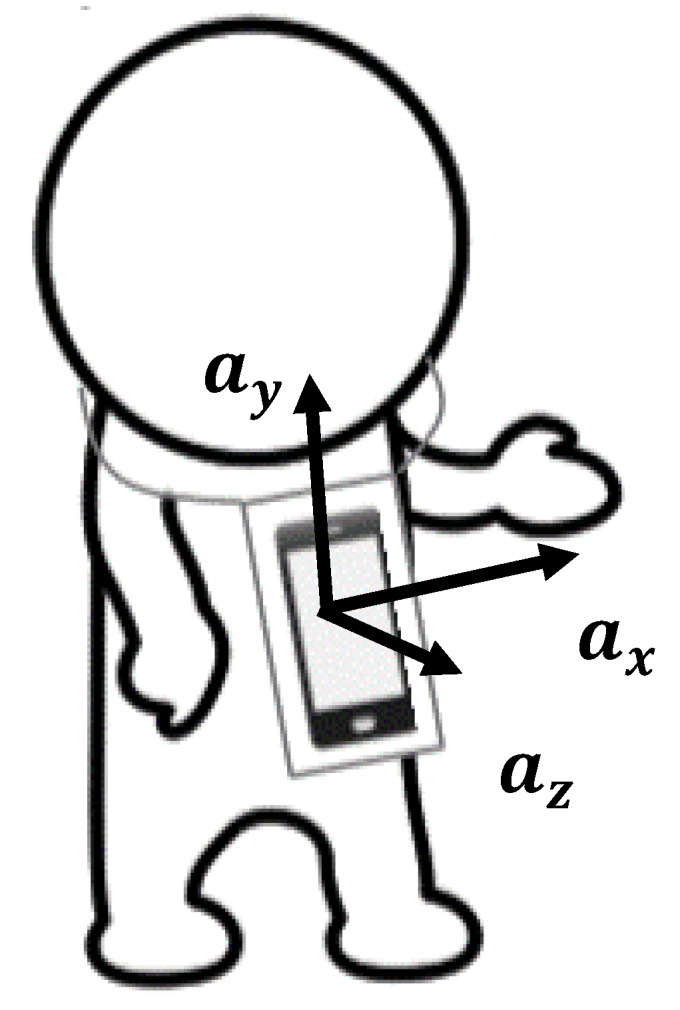
Smartphone sensor worn around the neck. The three axes of the built-in accelerometer are indicated by ($a_{x},\;a_{y},\;a_{z}$).

The utterances and physical motions of participants communicating in the participatory class were recorded using a smartphone (ZC551KL; ASUSTeK Computer Inc. (Taipei, Taiwan)) worn around their neck, which contained a built-in triaxial accelerometer capable of recording at a sampling rate of 100 Hz, and a microphone capable of recording at a sampling rate of 44,100 Hz (Figure 1). The recording duration was 60 min.

Students were divided into groups of four; each group sat in a circle on the floor and communicated amongst the group members. Approximately 190 students were in a seminar room of approximately 120 m^2^. The distance between groups was comparable to that between group members; thus, students were close enough to hear other groups’ conversations. Under an approximation that the students filled the room in a regular square lattice-like manner, the density translated into a mean interpersonal distance of 0.7 m.

To perform binary classification using supervised learning, all data were manually labeled as 1 (class speech) or 0 (class non-speech) in 0.1 s increments. This segment length was decided in consideration of the tradeoff between the effort required for manual labeling and the time resolution to be achieved. Typical speech durations are much longer than 0.1 s, and the length of our data is quite long (60 min), both contributing to statistical stability. Furthermore, most applications of VAD involve the extension of detected speech periods by a few hundred milliseconds on each side for stable results (the so-called hangover procedure) [6,26], lessening the need for higher time resolutions. Therefore, we deemed a segment length of 0.1 s to give sufficiently fine resolution for our purpose. Periods that could be identified as utterances were considered speech, as were those identified as laughter.

### 2.3. Data Preprocessing

Data preprocessing was conducted using MATLAB R2019a (MathWorks, Inc. (Natick, MA, USA)). 

#### 2.3.1. Audio Data

We adopted signal energy as the audio feature for speech discrimination, because it has been used as a primary audio feature in many VAD methods [6,8], can take advantage of wearable sensors (i.e., it is most sensitive to the wearer’s voice), and does not require personalized and complex modeling for each speaker. First, to reduce computational cost, the audio signal was down-sampled from 44,100 Hz to 11,025 Hz. Here, 11,025 Hz was determined in reference to the telephone audio sampled at 8 kHz. Second, a short-time Fourier transform (STFT) with a sliding Hamming window of 0.0232 s (256 samples) and 50% overlap (128 samples) was applied to represent the audio data in the time-frequency domain. Finally, by integrating the absolute square of STFT coefficients over frequencies and averaging at each 0.1 s time segment, we obtained power time series for use in the following analyses.

In addition, we evaluated the signal-to-noise ratio (SNR) of the audio data as follows. Based on the manual labels, we calculated the mean power during speech ${\bar{P}}_{\mathrm{speech}}$ and non-speech ${\bar{P}}_{\mathrm{nonspeech}}$ for each sensor. Then, the $\mathrm{SNR}=10\log_{10}({\bar{P}}_{\mathrm{speech}}/{\bar{P}}_{\mathrm{nonspeech}})$ indicates how much the signal level of the sensor wearer’s speech dominates the level of background noise, which includes the voices of other speakers. Table 1 shows the obtained SNR for the twelve participants (mean SNR = 8.55 dB). 

#### 2.3.2. Accelerometer Data

First, from the triaxial acceleration data $\left(a_{x}\left(t\right),\;a_{y}\left(t\right),\;a_{z}\left(t\right)\right)$ recorded using the wearable sensor, we calculated acceleration norm $a\left(t\right)=\sqrt{a_{x}^{2}\left(t\right)+a_{y}^{2}\left(t\right)+a_{z}^{2}\left(t\right)}$. Since the measurement is subjected to Earth’s gravity, the acceleration norm fluctuates around the gravitational acceleration of approximately 9.8 m/s^2^. Second, an STFT with a sliding Hamming window of 1.28 s (128 samples) and 1.18 s overlap (118 samples) was applied to represent the acceleration norm data in the time-frequency domain. Then, the power time series calculated by the absolute square of STFT coefficients at each frequency bin from 1 to 20 Hz with 0.1 s time steps were used as the physical motion features in the following analyses. The 60 min of power time series contained approximately 36,000 time points (excluding several time points at both edges where the sliding time window for STFT goes beyond the boundaries). Although activity classification studies using smartphone as wearable acceleration sensor often focus on lower frequencies (≤10 Hz) [21,25], we also included higher frequencies, as we could not eliminate the possibility that they contribute to speech discrimination.

### 2.4. Analysis to Verify Co-Occurrence of Utterances and Physical Motions

**Figure 2:**
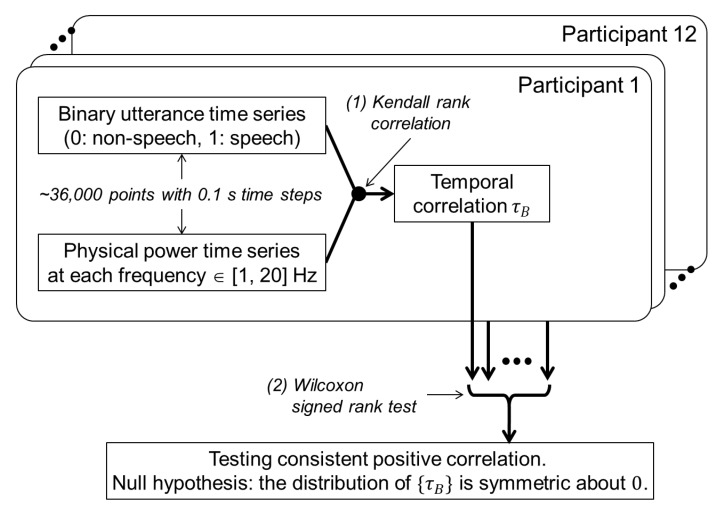
Analytic flow of the co-occurrence of utterances and physical motions.

To confirm the potential usefulness of the physical motion feature in discriminating speech vs. non-speech periods, we tested the co-occurrence relationship between utterances and physical motions, according to the following procedure (Figure 2).

At the individual level, we evaluated the degree of co-occurrence by calculating the temporal correlation between the power time series at each frequency (from 1 Hz to 20 Hz) of physical motion and the binary correct-answer time series (1: speech, 0: non-speech) for each participant (Figure 2 (1)). Here, we used nonparametric Kendall rank correlation, because the distributions of physical motion data are highly skewed (note that signal power is always positive) and prone to outliers. Since the binary utterance data have many ties, Kendall’s $\tau_{B}$(tau-b) was used to make adjustments for the ties [27,28]. Positive correlation indicates larger physical motion power during speech than non-speech periods. 

By performing a one-sample Wilcoxon signed-rank test on the correlation values at each frequency over participants (n = 12), we tested the consistency of the co-occurrence relationship between utterances and physical motions at the group level (Figure 2 (2)). For multiple tests over the 20 frequencies, family-wise error rate was controlled using the Bonferroni correction.

### 2.5. Utterance Discrimination within Individuals

In utterance discrimination within individuals, we performed analyses with the aim of verifying whether higher accuracy could be achieved by using both the audio and physical motion data, in comparison to only the audio data. 

The audio and physical motion power time series calculated, according to the preprocessing steps above, were used as the features. Each 60-min data contained approximately 36,000 samples corresponding to 0.1 s time segments. 

Using the random forest algorithm [29], for each individual data separately, we fitted and tested the three types of speech vs. non-speech binary classification models: (1) using only the audio feature, (2) using only the physical motion features, and (3) using both the audio and physical motion features. We fitted and tested the classification models using randomForest package [29] in R statistics software [30], with default parameters (ntree: 1000; mtry: 1 for model (1) and 4 for models (2) and (3); nodesize: 1; maxnodes: unlimited). As the data were measured in a participatory class in which students communicated in groups, the data could be non-stationary (e.g., the volume of voice and physical motion might have changed gradually as they became familiar with their groups). Therefore, we verified the accuracy of each classification model by a 10-fold cross-validation of randomly sampling without preserving the time series structure.

To address the classification of imbalanced data, the data balance was improved by generating positive example data (speech samples) and deleting negative example data (non-speech samples), using the synthetic minority over-sampling technique (SMOTE) [31] package in R. The method consists of a combination of over-sampling the minority class and under-sampling the majority class. The over-sampling of the minority class involves synthesizing minority class examples by joining existing minority class neighboring samples in the feature space. The method has been shown to help in constructing classifiers with better performance for imbalanced datasets [31]. The number of speech and non-speech samples before and after SMOTE for the twelve participants are shown in Table 2.

We evaluated the fitting of the three models using the accuracy, recall, and precision, with speech as positives and non-speech as negatives.

### 2.6. Utterance Discrimination over Individuals

We also tested the performance of the inter-individual prediction model, using both the audio and physical motion data in comparison to that of using only the audio data. This was to test whether the combined usage of the multimodal data can also benefit the utterance discrimination over individuals, where an existing model trained with a fixed set of data was desired to perform well for new data from new individuals, without requiring labor for manual speech annotation for the new data.

Here, we used the same audio and physical motion power time series as in the within-individual discrimination above. However, to alleviate the potential influence of individual differences in the characteristics of utterance and physical activities, the individual power time series were standardized using the median for centering and median absolute deviation (MAD) for scaling for robustness against outliers. Subsequently, these features were used for utterance discrimination over individuals.

Random forest classifiers with the three sets of features, (1) using only the audio feature, (2) using only physical motion features, and (3) using both the audio and physical motion data, were fitted and tested by leave-one-subject-out cross-validation, using the data of all participants. Here, of the data of twelve participants, the data of 11 individuals were used as training data, while the data of the other individual were used for the test at each fold. As with the utterance discrimination within individuals, the data balance was improved by over- and under-sampling, using SMOTE for imbalanced classification. 

Again, we evaluated the fitting of the three models using the accuracy, recall, and precision.

## 3. Results

### 3.1. Co-Occurrence of Utterances and Physical Motions

**Figure 3:**
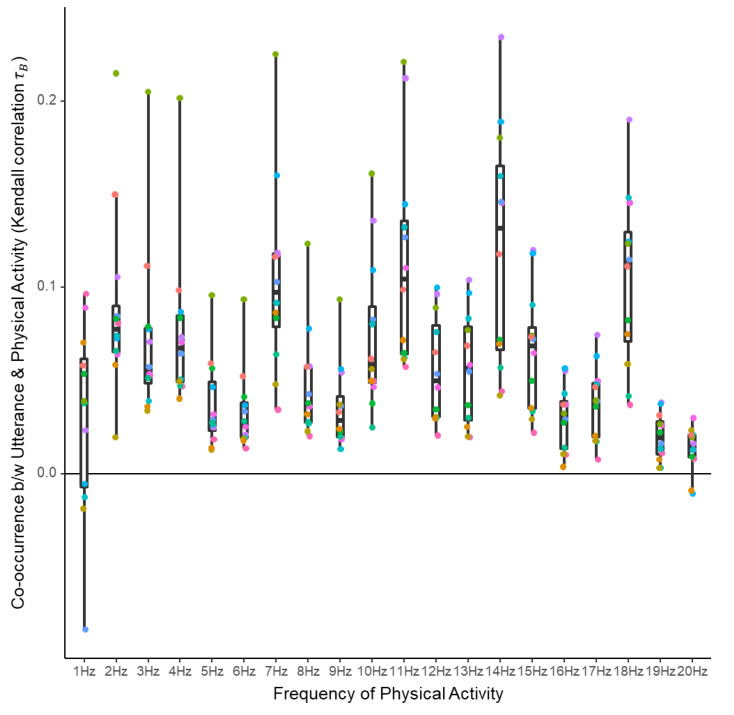
Kendall rank correlation coefficients indicating concurrent occurrence of utterances and physical motions at different frequencies. Colored points indicate different participants.

Temporal correlation analysis results that indicate the degree of co-occurrence between the utterance and physical motions at each frequency are shown in Figure 3. Although the correlation values were small and the sensitivity varied among participants, consistent positive correlation indicated a co-occurrence relationship between utterances and physical motions at an extensive range of frequencies from 1 Hz to 20 Hz. The consistency of the co-occurrence relationship was confirmed by one-sample Wilcoxon signed-rank tests on the correlation values over participants (Table 3).

### 3.2. Utterance Discrimination within Individuals

The results of the performance tests of utterance discrimination within individuals are shown in Table 4. In addition, we conducted paired t-tests to compare the average performance over individuals between the multimodal (combination of audio and physical motion) and other two models, and verified that the combination model performed significantly better in terms of accuracy (combination vs. audio only: t(11)=16.49, Bonferroni-corrected $p=2.5\times 10^{-8}$; combination vs. motion only: t(11)=16.79, Bonferroni-corrected $p=2.1\times 10^{-8}$), recall (combination vs. audio only: t(11)=15.27, Bonferroni-corrected $p=5.7\times 10^{-8}$; combination vs. motion only: t(11)=4.35, Bonferroni-corrected p=0.0069), and precision (combination vs. audio only: t(11)=14.25, Bonferroni-corrected $p=1.2\times 10^{-7}$; combination vs. motion only: t(11)=12.23, Bonferroni-corrected $p=5.8\times 10^{-7}$). These results confirm that a better discrimination could be achieved by using both the audio and physical motion data in all three performance metrics (accuracy, recall, and precision), compared to using only the audio or physical data.

### 3.3. Utterance Discrimination over Individuals

The results of the performance tests of utterance discrimination over individuals are shown in Table 5. As with the utterance discrimination within individuals, we also confirmed a higher performance of inter-individual prediction using both the audio and physical motion data in all three metrics (accuracy, recall, and precision), compared to using only the audio or physical data.

## 4. Discussion

To verify the hypothesis that a higher accuracy can be achieved in utterance discrimination by using physical activity data in combination with audio data acquired by wearable sensors, we performed experiments in a participatory class in which many students participate and speak at the same time. We tested the co-occurrence relationship between the utterance and physical motion and compared the performance of intra- and inter-individual prediction between the models that used multimodal and unimodal data.

First, the co-occurrence relationship between utterance and physical motion at a wide range of frequencies (1 Hz to 20 Hz) was confirmed from the analysis of temporal correlation between the manually identified utterances (correct data) and physical activities identified from the sensing data. This suggests that physical motions contain information about speech, and reinforces the validity of a previous study [21] that attempted utterance discrimination on physical motion features alone.

In the speech vs. non-speech binary classification, we confirmed a higher performance of intra- and inter-individual prediction by using both the audio and physical motion features, compared to using only the audio or physical motion features. This proved the effectiveness of utterance discrimination in a multimodal approach. As a related study for utterance discrimination using multimodal approach, there is a proposed system that combines audio features with video features. The system has been shown effective on datasets that contain not only constrained scenarios where the participants try not to move, but also dynamic situations such as people standing up and walking out of a room [14]. In comparison to such systems that utilize fixed microphone and cameras, our approach of utilizing wearable sensors has the advantage that it can be easily applied anywhere without the need to install fixed sensors, provided wearable sensors (i.e., smartphones) are owned by, or available to, the users. Similarly, a recent work on the audio-visual multimodal speaker diarization also discussed a potential extension by considering the use of wearable sensors to handle more complex scenarios involving tens of participants [32]. Meanwhile, the “wear-less sensing” approach using the fixed sensors also has its merits; thus, a complementary use or fusion of the wearable and wear-less multimodal sensing approaches are expected to be pursued in future studies.

Regarding the relatively lower accuracy of the inter-individual classification compared to the intra-individual classification, the impact of individual differences may be the major cause. There are various individual differences, such as in voice volume, frequency and temporal pattern of speech, and tendency toward moving actively or not when talking. In the intra-individual classification, where the training and validation data come from the same participant, such individual differences are handled by the prediction model customized for each person. However, the individual differences render it more difficult to identify the periods of speech in inter-individual classification, which learns the discrimination model with the data of specific individuals and applies it to the data of new and unknown individuals. In this study, individual differences in the scales of voice volume and magnitude of body movement were standardized separately. In future studies, incorporating the modeling and estimation of individual differences in the patterns of movement during speech could be helpful in improving the performance of inter-personal speech discrimination.

The purpose of this study was to verify the concept that multimodal sensing that combines audio and physical motion information can improve the performance of speech discrimination, compared to using only unimodal sensor data; hence, further studies are needed for an optimal classifier and feature selection. For example, we used random forest as the classifier, treating the data at each moment independently. To take the temporal structures in the feature data into consideration (e.g., by using hidden Markov model classifiers, as in previous studies [9,11,16,33,34]) can yield significant improvements in the stable identification of speech periods. Deep learning approaches have also been shown to be effective [35], and one study developed a convolutional neural network-based VAD algorithm that runs in real-time on a smartphone [36]. Regarding feature selection, we used only the one-dimensional intensity feature of audio data; however, the use of higher-dimensional features, such as mel-frequency cepstrum which has been widely used in speech recognition [14,15,37], and combining multiple features [26,38], can contribute to overall performance improvement. Integrating these more advanced state-of-the-art SAD/VAD methods with physical motion information available from wearable sensors can lead to even higher performance. Furthermore, to extend and generalize the effectiveness of our approach in various situations and with the different smartphone devices owned by individuals, we need to verify whether the high classification performance obtained in this study can also be achieved when using data from situations other than the target participatory class used in this study, and when using wearable sensors with different weights and sizes. The optimal wearing position of the smartphone sensor can also be refined.

In conclusion, this study has shown that the multimodal sensing of audio and physical motion during communication using wearable sensors is effective for improving utterance discrimination performance in real-world situations where many people participate and speak simultaneously. Further advancement of the method used in this study can enable a handy and widely available evaluation of communication in various dynamic real-world situations, where the number of participants and composition of a group are fluid, and many people speak simultaneously. Such technologies are expected to be useful in basic scientific studies to elucidate social processes and structures, as well as in engineering applications to provide support for improving efficiency, productivity, and soundness in a wide range of social groups and activities. Possible target fields in which communication is affected by noise include classrooms, office meetings, construction sites, hospitals, parties, public transportation, and other social activities.

## Figures and Tables

**Table 1:** Signal-to-noise ratio (SNR) of the audio data for the twelve participants.

	SNR (dB)
Participant 1	8.06
Participant 2	9.34
Participant 3	9.34
Participant 4	10.25
Participant 5	8.40
Participant 6	7.84
Participant 7	7.89
Participant 8	7.75
Participant 9	10.56
Participant 10	9.47
Participant 11	8.25
Participant 12	5.58

**Table 2:** Number of speech and non-speech samples (each corresponding to 0.1 s time segment) for the twelve participants.

	Original		After SMOTE	
	Non-Speech	Speech	Non-Speech	Speech
Participant 1	34,054	1934	7736	5802
Participant 2	30,464	5524	22,096	16,572
Participant 3	31,361	4627	18,508	13,881
Participant 4	32,583	3405	13,620	10,215
Participant 5	32,370	3618	14,472	10,854
Participant 6	32,466	3522	14,088	10,566
Participant 7	31,318	4670	18,680	14,010
Participant 8	33,004	2984	11,936	8952
Participant 9	33,713	2275	9100	6825
Participant 10	32,901	3087	12,348	9261
Participant 11	32,422	3566	14,264	10,698
Participant 12	32,809	3179	12,716	9537

**Table 3:** Results of one-sample Wilcoxon signed-rank tests (*V*-statistic, *p*-value, and Bonferroni-corrected *p*-value) on the co-occurrence between utterance and physical motion at each frequency.

Frequency	*V*-Statistic	*p*-Value	Bonferroni-Corrected *p*-Value
1 Hz	62	0.038574	0.77148
2 Hz	78	0.000244	0.00488
3 Hz	78	0.000244	0.00488
4 Hz	78	0.000244	0.00488
5 Hz	78	0.000244	0.00488
6 Hz	78	0.000244	0.00488
7 Hz	78	0.000244	0.00488
8 Hz	78	0.000244	0.00488
9 Hz	78	0.000244	0.00488
10 Hz	78	0.000244	0.00488
11 Hz	78	0.000244	0.00488
12 Hz	78	0.000244	0.00488
13 Hz	78	0.000244	0.00488
14 Hz	78	0.000244	0.00488
15 Hz	78	0.000244	0.00488
16 Hz	78	0.000244	0.00488
17 Hz	78	0.000244	0.00488
18 Hz	78	0.000244	0.00488
19 Hz	78	0.000244	0.00488
20 Hz	72	0.003418	0.06836

**Table 4:** Performance of intra-individual classification (%) using multimodal (combination of audio and physical motion) and unimodal data.

		Combination	Audio	Physical Motion
Participant 1	Accuracy	90.99	77.59	85.73
	Recall	88.90	68.52	83.31
	Precision	89.96	76.69	83.40
Participant 2	Accuracy	92.55	82.69	88.31
	Recall	89.24	74.80	86.90
	Precision	93.11	83.14	85.98
Participant 3	Accuracy	90.93	79.11	84.65
	Recall	87.56	73.11	76.77
	Precision	90.95	76.99	85.89
Participant 4	Accuracy	94.91	81.67	90.28
	Recall	94.38	76.74	93.20
	Precision	93.79	79.71	85.46
Participant 5	Accuracy	90.74	82.33	86.54
	Recall	85.91	72.13	80.15
	Precision	91.95	84.37	87.41
Participant 6	Accuracy	89.59	81.10	85.22
	Recall	84.59	70.92	76.69
	Precision	90.50	82.54	87.29
Participant 7	Accuracy	92.79	79.68	88.87
	Recall	90.47	69.65	88.11
	Precision	92.54	80.32	86.22
Participant 8	Accuracy	92.26	79.47	88.53
	Recall	90.69	69.50	89.58
	Precision	91.19	79.96	84.56
Participant 9	Accuracy	93.54	83.60	88.22
	Recall	90.84	76.02	88.48
	Precision	93.91	84.19	84.72
Participant 10	Accuracy	95.33	84.42	91.43
	Recall	94.00	76.83	92.72
	Precision	95.05	85.36	87.94
Participant 11	Accuracy	93.68	80.74	89.17
	Recall	92.23	72.29	88.97
	Precision	92.99	80.76	86.21
Participant 12	Accuracy	89.43	72.24	86.75
	Recall	87.14	60.08	85.04
	Precision	88.09	70.76	84.21
Average	Accuracy	92.23	80.39	87.81
	Recall	89.66	71.72	85.83
	Precision	92.00	80.40	85.77

**Table 5:** Performance of inter-individual classification (%) using multimodal (combination of audio and physical motion) and unimodal data.

	Combination	Audio	Physical Motion
Accuracy	83.22	67.66	71.86
Recall	74.23	72.81	59.02
Precision	84.50	60.09	70.02
